# Psychometric Properties of the Climate Change Worry Scale

**DOI:** 10.3390/ijerph18020494

**Published:** 2021-01-09

**Authors:** Alan E. Stewart

**Affiliations:** College of Education, University of Georgia, Athens, GA 30602, USA; aeswx@uga.edu

**Keywords:** climate change, worry, climate, psychometrics, psychological measurement, weather

## Abstract

Climate change worry involves primarily verbal-linguistic thoughts about the changes that may occur in the climate system and the possible effects of these changes. Such worry is one of several possible psychological responses (e.g., fear, anxiety, depression, and trauma) to climate change. Within this article, the psychometric development of the ten-item Climate Change Worry Scale (CCWS) is detailed in three studies. The scale was developed to assess proximal worry about climate change rather than social or global impacts. Study 1 provided evidence that the CCWS items were internally consistent, constituted a single factor, and that the facture structure of the items was invariant for men and women. The results from Study 1 also indicated a good fit with a Rasch model of the items. Study 2 affirmed the internal consistency of the CCWS items and indicated that peoples’ responses to the measure were temporally stable over a two-week test–retest interval (*r* = 0.91). Study 3 provided support for the convergent and divergent validity of the CCWS through its pattern of correlations with several established clinical and weather-related measures. The limitations of the studies and the possible uses of the CCWS were discussed. The current work represents a starting point.

## 1. Introduction

### 1.1. Climate Change and Mental Health

Global climate change continues to create physical, economic, health, and social impacts that require mitigation and adaptation [1]. From 2017 to 2019, there were 125 weather events worldwide that produced 943 billion US dollars in economic losses [2,3]. Methods and efforts have emerged to attribute severe and extreme weather events to a disrupted climate system [4,5]. Some of the most destructive events included tropical cyclones, inland flooding, and severe weather outbreaks [2]. In 2019, there were 4.1 billion people potentially exposed to natural hazards that included, predominately, weather- and climate-related disasters. Of these, there were 95 million people who were directly affected by these disasters [6]. In addition to these direct influences of the weather and climate upon people, global climate change has disrupted and destroyed ecosystems and thus threatened human, animal, and botanical habitats [7]. Climate change has also threatened supplies of food, water, and other resources, which has heightened the possibilities for inter-group conflicts [8,9,10].

The occurrence of global climate change has posed challenges to mental health [7,11,12,13,14,15,16]. Trauma, anxiety, fear, worry, and depression may accompany the short- or long-term effects of climate change [12,13,16,17,18,19]. Anxiety and fear may stem from the experience of extreme weather [20,21,22]. In addition, the impacts of climate change on ecosystems and, consequently, upon human cultures may underly feelings of dread and anxiety about the climate-related uncertainties that may be ahead [17,23,24,25]. Several researchers have documented worry about climate change and related it to political affiliation and support for climate change mitigation and adaptation policies [18,26,27,28,29]. People may experience reactions of grief, loss, and mourning following the changes brought by a disrupted climate; climate change may be the work of mourning [30,31]. A related concept is that of solastalgia which involves the experience of negative environmental change in a place that a person has known, become attached to, and/or inhabited [32,33].

### 1.2. Assessing the Psychological Effects of Climate Change

Several researchers have evaluated the stressful effects of degraded natural environments in general [34,35,36]. For example, Higginbotham et al. developed a measure of environmental distress that people experienced near an area of open mining in Australia [36]. Similarly, Bowler and Schwarzer studied the distressing experiences that people had in living near sites in New Mexico and Texas that involve exposure to toxic solvents. These researchers developed an environmental worry scale [35]. Other researchers, looking across multiple environmental risks, have identified several common dimensions of concern about the environment [34]. These involve: (1) impact upon species (nonhuman species), (2) possible benefits to humans, (3) impact upon humans, (4) avoidability (controllability), and (5) knowledge of the impacts (observability/predictability). Using these dimensions along with a consideration of time, Böhm provided a classification of the negative emotions that people may experience related to the environment risks [37]. For environmental consequences that have already occurred (retrospectively), for example, people may feel regret, sadness, or sympathy among other emotions. For negative consequences that may yet occur (prospective timeframe), people experience fear, worry, or hopelessness, among other emotions [37]. Such prospective consequentialist emotions encompass the feelings that may arise as people anticipate the effects of climate change. Specifically, anxiety and worry have become the focus of research on the psychological effects of climate change.

#### 1.2.1. Climate Change Anxiety

Climate change anxiety has emerged from research on eco-anxiety and the psychological effects of climate change [13,25]. Eco-anxiety, which has been defined as “a chronic fear of environmental doom” ([16], p. 68). One researcher defines climate anxiety as “anxiety which is significantly related to anthropogenic climate change” ([25], p. 3). Clayton similarly defined climate change anxiety as “anxiety associated with perceptions about climate change, even among people who have not personally experienced any direct impacts” ([23], p. 2). Clayton and Karaszia created a 13-item scale for assessing climate change anxiety. This instrument consists of eight items measuring cognitive-emotional impairment (e.g., thoughts about climate change and its effects on concentration, sleep, nightmares, crying, and coping) and five items measuring functional impairment (e.g., climate change concerns affect relations with friends and family, ability to complete school or work) [24]. With these items, the researchers appeared to be assessing more severe or impairing levels of anxiety about climate change. Beyond developing their scale, the authors observed that climate change anxiety was correlated significantly with participants’ emotional responses to climate change [24].

#### 1.2.2. Climate Change Worry

Several researchers have examined climate change worry in relation to other psychological or demographic variables [18,26,27,28,29,38]. In some of these investigations, the researchers have studied concern about climate change and have operationalized concern through items that inquire about the degree of worry that people reported [29,38]. Gregerson et al. observed within the European countries that political orientation and expectations of negative impacts most predicted climate change worry [26]. Similarly, views of climate change and policy preferences to address it were predicted by self-reported degree of worry [18]. Another research team surveyed France, Germany, Norway, and the United Kingdom in order to examine similarities and differences (among other things) in the concern/worry among the four nations [38]. A study of children and adolescents in Sweden examined ways that people cope with worry about climate change and can find ways to be hopeful for the future [28]. Researchers in Australia assessed worry and concern about climate change and observed correlations of such worry with symptoms of stress, anxiety, and depression [29]. Finally, another team of researchers observed that knowledge of the causes and consequences of climate change predicted worry about climate change [27].

Three points emerge from the consideration of the aforementioned studies, the first of which is that climate change worry (sometimes discussed as concern) has been the of interest to a number or researchers. Second, and important for the present project, all six of the studies cited above measured climate change worry though the use of a single item or repetitions of a single item. In his discussion of the measurement of climate change risk perception and worry, van der Linden and others have recommended against the use of single-item measures of a construct [17,39,40]. Third, items that focus on global or general societal worry (concern) about climate change may not relate as reliably to personal experiences, psychological processes, behaviors, and/or policy preferences as items that assess personal worry about climate change [41]. With these points in mind, researchers may benefit from the creation of a brief, multi-item measure of personal worry about climate change.

The author’s purpose in this article is to describe the psychometric development of the Climate Change Worry Scale (CCWS), a ten-item self-report measure designed to assess the level of troubling, disturbing thoughts that people experience about climate change. This article begins with a discussion of worry and how it differs phenomenally from the related constructs of anxiety and fear. The author then describes the construct of climate change worry along with the procedures used in the development of the CCWS. In three studies that follow, the author details the psychometric development of the CCWS. Study 1 presents the results of the factor analysis of the items and assesses its internal reliability. This study also investigates the invariance of the latent structure of the measure with respect to gender and also presents the results of a Rasch modeling analysis of the items. Study 2 presents results on the test–retest reliability of the CCWS. Finally, Study 3 provides results about the convergent and divergent validity of the CCWS through its pattern of correlations with several established clinical and weather-related measures.

### 1.3. The Constructs of Worry and Climate Change Worry

Borkovec and colleagues provided an initial definition of worry that has generally been supported by empirical research over time: “Worry is a chain of thoughts and images, negatively affect-laden and relatively uncontrollable; it represents an attempt to engage in mental problem-solving on an issue whose outcome is uncertain but contains the possibility of one or more negative outcomes; consequently, worry relates closely to the fear process.” [10,42].

Subsequent to this definition and with the creation of the Penn State Worry Questionnaire, Borkovec and colleagues emphasized worry as encompassing negative verbal-linguistic thoughts and minimal amounts of imagery [43,44]. Worry also involves apprehensiveness about future negative events [45,46]. The experience of worry as an emotional state is associated with the tension, nervousness, irritability, and difficulties in remaining calm, all of which are characteristics of stress [47,48]. The distinguishing characteristics between normal worry versus persistent, problematic worry is that the latter involves worry about a wider variety of concerns, that episodes of worry occur more frequently in time and last longer, and that the worry is experienced as repetitive and uncontrollable [45,49,50]. Further, while normal and adaptive worry involves attention to a threat in the environment and provides resources for thinking and problem solving, excessive and maladaptive worry may involve difficulties in disengaging from repetitive thinking such that problem-solving abilities around the threat are, paradoxically, compromised [51].

How does worry differ from fear, anxiety, and depression [52,53,54] Fear involves the appraisal of something as an immediate, emergent threat coupled with a high degree of somatic arousal that is mobilized in service of either fighting the threat or fleeing from it [54]. Fear is an immediate and intense emotional response that is adaptive in responding to a threat. Anxiety encompasses both worry and somatic, autonomic arousal. Thus, anxiety as a basic emotion involves repetitive verbal-linguistic thoughts of apprehensive events and the bodily symptoms of increased heart rate, respiration, sweating, and so forth [47,51,54,55]. Unlike fear, anxiety is a response to scenarios that pose more diffuse threats in the future [51]. Anxiety about perceived threats may generalize across situations, settings, and people such that a person experiences General Anxiety Disorder [51]. Depression also involves worry but includes additional features of sadness, despondency, a negative view of the self, and a sense of hopelessness for the future [47]. Worry and negative, uncontrollable thinking may be constituent core processes of both anxiety and depression [44,56].

With these distinctions in mind then, the construct of climate change worry involves primarily verbal-linguistic thoughts (rather than images) about the changes that may occur in the climate system and the possible effects of these changes. These thoughts may be persistent, repetitive, or difficult to control. Climate worry could involve a focus upon increases in the severity of the weather. Alternatively, climate change may require both coping and adaptation responses, the unknown nature of which may be a focal point for worry. People also may worry about the effects of climate change upon others, to habitats, lifestyles, livelihoods, health and so forth [22,23]. In moderation, worry may help a person to adjust and adapt to a changed climate [17,41,57]. Excessive worry, however, may preclude efforts to adapt and result in tension, distress, and decreased abilities to solve problems [42,45]. Such elevated levels of worry also may contribute to other emotional challenges like anxiety or depression [51,55]. Although people may become anxious about climate change as described by other researchers [24], the focus of the present research is upon measuring the degree of worry that people may experience about climate change.

### 1.4. The Climate Change Worry Scale

The author developed the CCWS items to assess the frequency of worry as it relates to the domain of a disrupted, changing climate. To maintain a focus on the phenomena of personal worry rather than anxiety, fear, or depression, all but one item (i.e., #3) contains the term, worry, usually used as the main verb in the item statement that pertained to the respondent’s own experiences or behaviors [17]. Similarly, each item also uses the terms “climate change” or “changing climate”. The items require essentially require people to self-report their experiences of worrying about climate change.

The content for the items was informed by the climate psychology and worry literatures reviewed above. The author chose to focus item content on possible proximal manifestations of climate change such as severe weather outbreaks and effects upon others the respondent cares about rather than upon impacts such as inter-group conflicts or resource scarcity. There were two reasons for this decision, the first being that the author desired to keep the measure brief and to focus on a single construct of personal worry about climate change rather than worry of general, societal, or global origins. This was a deliberate choice because personal worry “is an active emotional state that is often closely linked to adaptive behavioral responses aimed at reducing a particular threat, whereas broad concern is not and can be expressed without any particular motivational or emotional content” [17,18,23,41,42]. Second, a focus on personal worry about climate change is consistent with research suggesting that people can personally experience proximal impacts of climate change and that such experiences are good predictions of climate change risk perceptions [20,41,58]. Thus, the CCWS items are limited to proximal and personal worry about climate change. This represents a starting point in assessing peoples’ experiences of worry.

The items of the CCWS appear in Table 1 along with the citations to sources in the reference list that informed or inspired their inclusion in the scale. Items 1, 2, and 8 assess the respondent’s phenomenological experience of worry about climate change; item 1 has the respondent comparing him or herself with others regarding the degree of worry. Items 2, 4, and 10 make reference to a future timeframe and worry about what a changed climate may bring. Item 5 references outbreaks of severe weather as possible indicators or markers of climate change. This item assesses worry about weather events that are more immediate or proximal in time. Items 3, 6, and 7 pertain to different attempts to respond or cope with climate change worry, one of which (item 3) involves the gathering of additional information. Items 6 and 7 each pertain to worry about being able to respond to climate change or feeling unable to cope with climate change. In addition, items 6, 7, and 9 were intended to tap the feature of uncontrollably worrying about climate change. Because research on worry has suggested that it is an episodic phenomenon that can vary with respect to frequency in time, the author chose a five-point frequency rating scale for people to use in responding to the items [19,25].

### 1.5. CCWS Development Procedures

Beyond the use of the literature in climate psychology and worry to develop the CCWS items, the author followed the recommended scale development procedures to examine the functionality of CCWS items [59,60,61,62]. Factor analysis is a tool for establishing construct validity of the items within a measure [59]. From the perspective of invariant measurement, Rasch analysis examines the relationships of the items in the scale with the amount of the characteristic (here, climate change worry) that people possess; both items and respondents are modeled [60,63,64]. These procedures were followed in Study 1. Another important psychometric to establish is the temporal stability of the scores that people generate on a measure [59,61]. For the construct of climate change worry, the author expected that levels of such worry would be relatively stable; the author examined the CCWS test–retest reliability in Study 2. Finally, the usefulness of a new measure can be demonstrated through its correlations with other known and established measures [59,60,61]. The author examined CCWS correlations with other measures in study 3 in following the procedures for assessing convergent validity.

## 2. Study 1: Factor Analysis and Rasch Model

The author’s purpose in the first study was to evaluate the psychometric properties of the of the ten CCWS items as given in Table 1. In addition to item descriptive statistics and an evaluation of the internal consistency of the items, the author also assessed the items’ contributions to the latent variable of climate change worry. Two approaches were used for the for the latter task: (1) the classical true score test theory approach involving factor analysis [59,64] and (2) a more contemporary approach to invariant measurement using a Rasch model [60,63,65]. In using each approach for studying the item relationship with the construct of climate change worry, the author was interested in evaluating the unidimensionality of the items (i.e., is a single latent variable responsible for the intercorrelations of the ten items?). The author also was interested in assessing the extent to which the items may function invariantly with respect to the respondent’s gender. Similarly, the author explored gender differences on mean CCWS scores.

Finally, the author examined the correlation of CCWS scores with scores on an item that assessed the participant’s liberal versus conservative political orientation. Although the IPCC has documented that the Earth’s climate is changing and that this has stemmed from anthropogenic forcing of the climate system [1], people differ with respect to the ways that mitigation and adaption efforts should proceed [66,67]. Political identifications and attitudes particularly relate to: the degree to which people believe that climate change and disruption is actually occurring, that climate change is a problem that should be addressed, and in beliefs about mitigating climate change and adapting to it [66,67]. Specifically, Smith and Leiserowitz observed that greater worry about climate change was associated with support for policy recommendations to address climate change [18].

### 2.1. Materials and Methods

#### 2.1.1. Participants

The participants were 600 undergraduate and graduate students from a large public university in the southeastern United States; the sample contained 300 each of men and women. The sample ranged in age from 18 to 51 years; *M* = 22.3 years and *SD* = 5.9 years. The racial composition of the participant sample was: 68.0% White, 10.0% Asian, 4.3% Black, 2.7% Latinx, 2.5% Indian subcontinent, and 12.5% multiracial or other race. Politically, 50.2% of the respondents indicated that they were liberal or liberal leaning and 35.9% were conservative or conservative leaning. Of the remaining participants, 9.4 indicated that they were moderate (between liberal and conservative) and 8.2% did not indicate their political orientation. The participants were recruited via an email message that was sent to the student that described the research project, which was part of a larger effort to gather data on the psychology of weather and climate. The incentive for participating was entry into a random draw to receive one of four $50 amazon.com gift cards. Students also had the chance to win one of the cards without participating in the research.

#### 2.1.2. Measures

The participants completed the CCWS items online using the Qualtrics survey platform (http://www.Qualtrics.com). They also completed a 12-item weather history survey that inquired about their past experiences of severe weather (i.e., floods, tornadoes, hurricanes, winter storms, or thunderstorms) that resulted in either injuries or damage to property. This survey also inquired about their previous evacuation experiences due to floods or hurricanes. The response options for this survey were “yes” or “no”. The online presentation order of the CCWS and the weather history survey was randomized. Finally, the participants then responded to demographic items that inquired about their age, gender identification, and race. The participants then responded to a single item that asked them to use a 7-point fully anchored rating scale to indicate their political orientation on a liberal (left side of scale) to conservative (right side of scale) scale.

#### 2.1.3. Procedure

The students were sent an email inviting their participation in the research and that also provided a link to the survey in Qualtrics. The first page of the online survey consisted of the informed consent document. If participants agreed participate, then the project measures appeared in random order. The demographics items always appeared last in the survey, followed by a debriefing statement. The project measures and procedure were reviewed and approved by the Institutional Review Board at the author’s university (Approval: MOD00007528, Parent Protocol STUDY00001233).

#### 2.1.4. Data Analysis

The author used both (1). factor analysis and structural equation modeling (SEM) approaches and (2). Rasch modeling to examine the latent structure of the CCWS items and to examine any differences in the functioning of the items with respect to participant gender. The author employed each of these psychometric approaches to provide a thorough examination and analysis of the items [64]. Response scales such as the one employed on the CCWS often function as ordered categories rather than a true interval-level scale. This means that item intercorrelations are best assessed through polychoric correlations [68,69]. The author used both the Lavaan package in R and the Factor program to calculate the polychoric correlations and to perform the exploratory factor analysis [70,71,72,73,74]. Factor is an open-source program available at: http://psico.fcep.urv.es/utilitats/factor/index.html. The factor analysis used an unweighted least squares factor extraction procedure because this method produced superior results compared to maximum likelihood methods when factor analyzing categorical data that are not normally distributed [75]. Because a single latent variable (i.e., climate change worry) was expected in the analysis of the data, no factor rotation methods were employed. The author used the Coefficient Alpha package in R to calculate the internal consistency statistics [76]. To check for the invariance of factor solutions across gender, the author used the Lavaan structural equation modeling package within the R statistics environment [74]. The semTools R package also was used to evaluate the extent of model invariance between men and women as constraints were added progressively to the models [77].

While traditional factor analysis is concerned with understanding the variance that items share in indicating a latent variable, Rasch modeling goes further to provide a description of both the items as well as the people who have responded to them (60,63,65). The goal of Rasch measurement is to create an invariant measurement model by calibrating responses to items with estimates of the amount of trait or ability a person possesses. For this project, the author used the Facets and Winsteps programs to perform the Rasch modeling [78,79].

### 2.2. Results

#### 2.2.1. Descriptive Statistics

Table 2 shows the polychoric correlations of the combined sample of men and women who responded to the CCWS. The bottom rows of Table 2 also provide the descriptive statistics for each item and the item-to-total correlations. The items exhibited a mix of positive and negative skewness. Overall, many items exhibited heavier distributions in the tails (i.e., negative kurtosis). These results were reflected in Mardia’s tests for multivariate skewness (not statistically significant) and kurtosis (*p* < 0.0001) [80]. Thus, the data were not considered to be normally distributed.

#### 2.2.2. Factor Analysis

Prior to conducting the factor analysis, the author examined Bartlett’s test of sphericity that the polychoric correlations in Table 1 were an identity matrix (i.e., 1’s on the diagonal and zero values elsewhere). Bartlett’s test (χ^2^ = 6673.2; df = 45; *p* = 0.00001) suggested the correlations were not an identity matrix, indicating that factor analysis was appropriate. The author also examined the Kaiser–Mayer–Olkin (KMO) test that at least one latent variable may explain the correlations in Table 1. The KMO value of 0.95 suggested that factor analysis would be useful. Several indices also suggested that a single factor existed among the item, beginning with the parallel analysis criteria of Timmerman and Lorenzo-Seva [81]. Three additional indices that are calculated by the Factor program also suggested a single, unidimensional factor. First, the unidimensional congruence (UniCo) coefficient was 0.994 (95% CI: 0.991–0.997). Second, the explained common variance (ECV) coefficient was 0.926 (95% CI: 0.909–0.942). UniCo and ECV values that are greater than 0.95 and 0.85, respectively, suggest that items can be treated as unidimensional. Third, the mean of item residual absolute loadings (Minreal) provides an indication of additional variance that was unexplained. The Minreal index was 0.223 (95% CI: −0.196–0.245), which was below the cutoff of 0.30, suggesting that the mean residual loadings were minimal. Given the values of these indices, a one-factor solution was chosen in the factor analysis.

Table 3 shows factor loadings of the items and their communalities. The single factor explained 73.6% of the variance. Likely due to the larger sample size, the robust mean and variance adjusted chi square statistic was statistically significant, suggesting a lack of model fit, χ^2^ = 302.72; df = 35; *p* = 0.00001. The root mean-square error of approximation (RMSEA) was 0.043, which indicated a well-fitting model. Beyond this, several fit indices suggested an acceptable degree of fit: the non-normed fit index = 0.97, the comparative fit index (CFI) = 0.99, and the adjusted goodness of fit index = 0.99. Finally, the root mean square of residuals was small (0.052), suggesting that the one-factor solution was optimal.

With respect to the internal consistency and unidimensionality of the CCWS items, Cronbach’s alpha was high; α = 0.95, (95% CI: 0.95–0.96) [82]. Because McDonald’s omega (ω) provides a more conservative estimate of internal consistency, the author also calculated this statistic as well [83,84]. Here, McDonald’s omega also exhibited a high value, ω = 0.95, (95% CI: 0.95–0.96). Hancock’s index, H = 0.97 (95% CI: 0.96–0.97), was calculated and suggested that a single common factor well-represented the CCWS items [85].

To what extent is this factor solution invariant for men and women in the sample? Table 4 shows the changes in the chi square, the CFI, and RMSEA as model constraints were added to assess the degree of invariance that may exist in the factor solution between men and women. Generally, the results of the invariance analyses support the conclusion that the CCWS items function very similarly in the assessment of climate change worry regardless of gender. Using a criterion of 0.005 for the change in the CFI and the RMSEA, the constrained equivalence of factor loadings (metric invariance), intercepts (scalar invariance), and the residuals of items (residual invariance) did not result in large changes in the model fit indices [86,87]. Thus, these types of invariance in the CCWS measurement model were established for men and women. The only criteria that was not satisfied concerns the constraint of the means of each group on the latent factor of climate change worry. Mean differences on the CCWS scores will be examined further below.

#### 2.2.3. Rasch Item Analysis

The Rasch model explained 71.3% of the variance in the CCWS items. The CCWS exhibited a good separation of the participants, *rel_part._* = 0.92, χ^2^ (599) = 6959.3, *p* < 0.0001. Such a reliability value suggests that the CCWS items are sensitive to the differences that participants exhibited on the latent trait of climate change worry. The Rasch analysis also provided a strong degree of separation of the items with respect to their distinctiveness and to their ordering as participants responded to them, *rel_item_* = 0.99, χ^2^ (9) = 2921.1, *p* < 0.0001.

**Figure 1:**
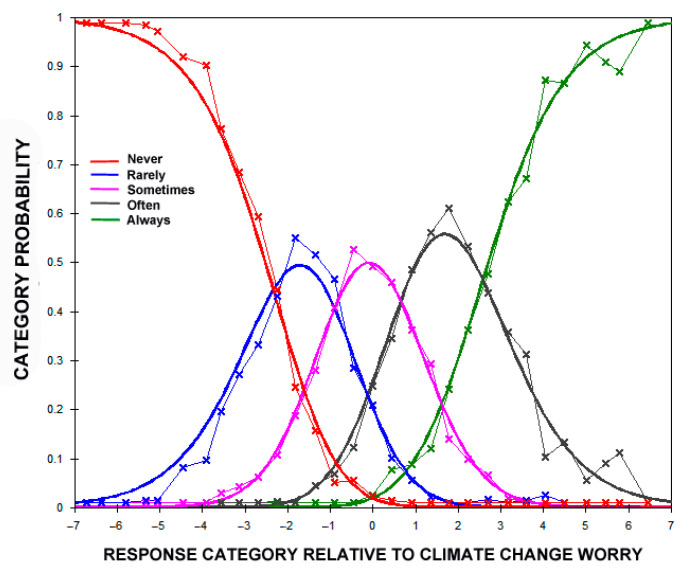
Actual and modeled proportion of responses within each response category. Note: x represents data and smooth lines represent the Rasch model.

Table 5 shows the frequency with which the different ordinal response categories were used by the 600 participants in responding to the CCWS items. Respondents used the first four categories rather uniformly (i.e., 21%–25%); the Always response category was used somewhat less at 11% overall. The average measures are those that were modeled to generate the observations at each response category. The expected measures are those corresponding response values that exist if the data fit the Rasch model [79]. The small discrepancies between the average and expected measures in Table 5 suggest that the data fit the Rasch model well. Similarly, the outfit column in Table 5 has an expected value of 1.0 for each category if the data fit the model. Finally, the Rasch–Andrich thresholds show the step calibrations of the rating scale as logits on the latent variable of climate change worry [79]. The thresholds show the amount of worry needed to go from one point on the response scale to the next (e.g., a 1.55 logit step is involved in moving from the Sometimes to Often response categories). Each of the thresholds in Table 5 exceeds the recommended minimum value of 1.4 logits [60]. Figure 1 shows the actual and the modeled proportion of responses within each response category of the CCWS items given the level of climate change worry, modeled as a latent variable. The close correspondence between the data (straight lines with x) and the model (smooth solid lines) provide visual support of the extent of Rash model fit.

Table 6 shows the Rasch model item characteristics along with statistics that indicate the degree to which the data fit the model for each item. The construct level (also known as the item difficulty) convey in logit units the level of climate change worry that the item assesses. The larger the value of the construct level, the greater the amount of climate change worry a person must have to respond to the item that they *often* or *always* worry. The modeled standard error appears in the second column of Table 6. The item discrimination coefficients appear in the third column of Table 6. These coefficients indicate the slope of the item characteristic curves and convey the degree to which items discriminate between people who possess different levels of climate change worry. The right columns of Table 6 suggest that the data fit the Rasch model well because the values for the infit and outfit mean squares were in the optimal region of 0.5 to 1.5 [79]. The infit statistic is sensitive to aberrant response patterns within the measurement region of an item whereas the outfit statistic is sensitive to outlying data points that do not fit the model well [60,79].

**Figure 2:**
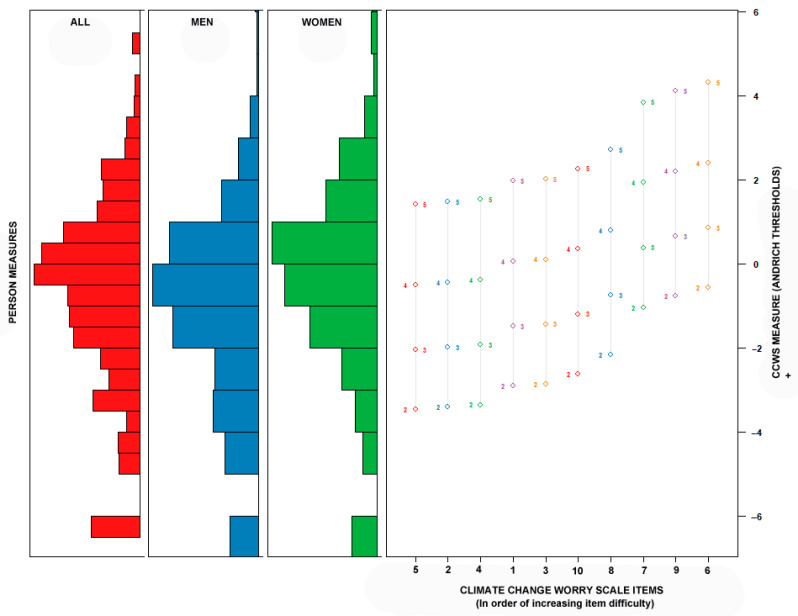
Wright map of the Climate Change Worry Scale item.

Figure 2 depicts a Wright map of both the items and the respondents on the logit scale of climate change worry [78,88]. The items and their response categories have been indicated on the right-hand side of the figure in order of increasing climate change worry (i.e., item difficulty). Generally, the ten CCWS items provide good coverage over the mid- and upper-ranges of climate change worry. Item coverage of lower and minimal levels of climate change worry was somewhat sparse.

**Figure 3:**
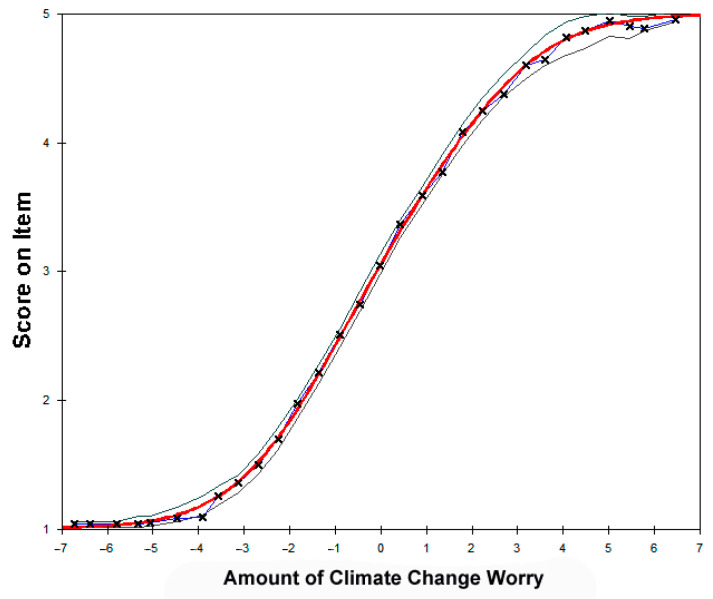
Test characteristic curve for the ten CCWS items.

Figure 3 shows the test characteristic curve for the ten CCWS items. The amount of climate change worry as a latent variable is depicted along the horizontal axis while the item response categories are shown on the vertical axis. The data fit the model well, as shown by the close proximity of the data (x) with the model (smooth curve). The figure also shows the upper and lower bounds of a 90% confidence interval. Overall, the figure suggested that the data fit the model quite well.

#### 2.2.4. Demographic Relationships with CCWS Scores

The author transformed the Rasch score on the CCWS for each person to remove the negative values (i.e., multiplied the Rasch score by 5 and added 50). When using four categories of the participants’ racial identification (Black, White, Asian, and Other), no statistically significant differences were observed in the mean of each group on the CCWS; *F* (3595) = 1.54, *p* = 0.20. The respondents’ political orientation scores were correlated significantly with their scores on the CCWS; *r* = −0.45, *p* < 0.0001. That is, self-ratings of being politically liberal were associated with higher scores on the CCWS whereas greater identification with conservative attitudes was associated with a lower score. Finally, the author examined the extent to which women and men differed in the mean scores on the CCWS given the relationships the CCWS scores demonstrated with political orientation. Women exhibited significantly higher scores (*M* = 47.6, *SD* = 12.1) compared to men (*M* = 45.4, *SD* = 11.8); *F* (1548) = 5.63, *p* = 0.02, η^2^ = 0.02. Overall, both political identification and gender accounted for 21% of the variance in CCWS scores. Finally, there were no statistically significant relationships among the CCWS score and the total or individual item responses to the weather history survey.

### 2.3. Discussion

The results from Study 1 were useful in establishing the psychometric properties of the CCWS items in several ways. First, the ten CCWS items appear highly internally consistent with respect to the way that the study participants interpreted and responded to them. Second, and relatedly, the factor analysis of the polychoric correlations suggested that a single latent variable, climate change worry, fit the data well. Third, the results of a confirmatory factor analysis provided support that even with constraints through the equality of residuals, the factor structure of the CCWS is invariant between men and women in the sample. Fourth, Rasch modeling provided further psychometric information at the item and person levels regarding the use and performance of the items and the five-point rating scale. In this regard, the items provide good coverage in reflecting the levels of climate change worry people possess. There is, however, a lack of items measuring very low or minimal levels of climate change worry. The demographic analyses revealed that the mean scores by men and women on the CCWS differed and that climate change worry was moderately correlated with liberal–conservative political identification. Further, prior experiences of severe or extreme weather that resulted in property damages or injuries were not associated with scores on the CCWS. This result was consistent with prior research [66].

## 3. Study 2: Test–Retest Reliability

The author’s purpose in the second study was to evaluate the stability of the CCWS scores over time when the scale was administered to a sample of people over two occasions. The observation of highly related scores over time provides evidence that CCWS may function similarly when used with groups that resemble the reliability sample [59,89].

### 3.1. Materials and Methods

The material for this study consisted of printed, hard-copy packet that contained the CCWS and a demographic form. The participants were 54 (45 women) undergraduate students from a large university in the southeastern United States. The mean age of the participants was 20.9 years (SD = 1.06 years). The racial composition of the participant sample was: 63% White, 24% Black, 6% Asian American, and 7% other, biracial, or multiracial.

Participation in the research fulfilled the students’ course requirements. The participants had a number of different research and non-research opportunities from which to choose. The 54 participants completed the research in person in groups of five to ten people each. When the participants registered for the research, they also were scheduled for their second session that was exactly two weeks after the first session. At the first session, the participants were provided with an overview of the research and completed a written informed consent document. They then completed the CCWS and were excused. At the second session, participants again completed the CCWS and were then provided with a written debriefing statement that summarized the purposes of the research. Care was taken at the first session to minimize the possibility of carry-over effects to the second session. The research protocol was part of a larger effort to evaluate the test–retest reliability of several weather and psychology measures. The research protocol was approved by the Institutional Review Board of the university where the author conducted the research (Protocol ID#: STUDY00003846).

### 3.2. Results

The Pearson correlation of the participants’ total scores on the CCWS at the first administration with their total scores at the second administration suggested that the sample produced reliable scores; *r* = 0.91, *p* < 0.001. As another check on the stability of the scores over the two-week test–retest interval, the author conducted a paired t-test of peoples’ scores on the CCWS on the two testing occasions. This analysis revealed that the CCWS scores also did not change in a statistically significant way from the first administration (*M* = 22.26, *SD* = 6.98) to the second administration (*M* = 22.89, *SD* = 7.00), *t* (53) = 1.53, *p* = 0.13.

Beyond this test–retest reliability assessment of scores, the author also evaluated the internal consistency of the CCWS items at the first and second administrations using Cronbach’s coefficient alpha (α). At both the first (α = 0.90) and second administrations (α = 0.91), the CCWS items demonstrated good internal consistency. Regarding McDonald’s omega, both the first administration (ω = 0.90) and the second administration (ω = 0.92) of the CCWS suggested that peoples’ responses to the items were internally consistent.

### 3.3. Discussion

The results of Study 2 provided evidence in two ways that respondents’ total scores on the CCWS were stable over time. First, when the respondents’ scores were correlated across a two-week test–retest interval, other than the passage of time the scores shared 83% of the variance. Second, the respondents’ mean scores were compared across the two occasions and did not differ with respect to their location (i.e., mean values). Additionally, with a new sample beyond that used in Study 1, the CCWS items demonstrated satisfactory internal consistency. Thus, the results from Study 2 establish the important psychometric property of temporal stability in the CCWS scores [59].

## 4. Study 3: Relationship of CCWS Scores with Measures of Worry, Fear of Weather, Anxiety, Depression and Stress

Having established the unidimensional structure of the CCWS items and the temporal stability of peoples’ responses to the measure, the author’s purpose in Study 3 was to explore the relationship of respondents’ scores on the CCWS with their scores on several related measures. Such a survey of correlations with known and established measures would permit an evaluation of the CCWS’s convergent and divergent validity. With respect to convergent validity, it was expected that as a measure of worry the CCWS would exhibit some degree of correlation with the Penn State Worry Questionnaire (PWSQ), a broad-based measured that inventories the degree to which people report worry in their lives generally [43,44]. Because the CCWS was designed to assess personal worry specifically about climate change rather than about broader areas of life, the correlations with the PSWQ were not expected to be large.

Because worry can be related to fear, the author expected that scores on the CCWS would be related to two measures of fear about weather conditions. Conceptually, if climate change manifests as future severe weather events that may be more intense and damaging, then worry about climate change should be related to fears about the weather [3,4,5]. In assessing the CCWS’s convergent validity with respect to fear, the author used two measures, the first of which was the Storm Fear Questionnaire (SFQ), a 15-item measure that was created to assess clinically-severe levels of storm fear in people [57]. The second measure was the Fear of Weather Scale (FOWS), an 87-item instrument designed to assess the degree of fear people experience towards various types of weather events and the consequences of these events for their lives [22].

The author examined the CCWS scores with those of stress, anxiety, and depression as measured by the Depression, Anxiety, and Stress Scale (DASS) [90,91]. The DASS has become a benchmark resource in defining, understanding and assessing the emotional states of depression, anxiety, and stress. For this study the author hypothesized that the CCWS would exhibit a greater degree of correlation with the DASS stress subscale, and somewhat lower correlations with the anxiety and depression subscales [45,46,49]. Further, the author also expected that anxiety would be related to scores on the weather fear measures and thus relate to CCWS scores more indirectly.

Beyond the pairwise correlations of the aforementioned measures with the CCWS, the author examined the extent to which the measures may be used together to account for CCWS scores. From Study 1, it was expected that peoples’ political orientations would be a significant predictor of climate change worry. The author predicted that stress would also be associated with climate change worry given the theoretical relationship of stress with worry [52]. Because fear of severe and extreme weather events may encompass what people would anticipate under a changed climate, it was expected that such fears also would be associated with CCWS scores. Finally, the author hypothesized that the emotional state of anxiety, rather than being directly predictive of climate change worry, would be more related with fear of weather and storms and thus exert its influences in that manner.

### 4.1. Methods

#### 4.1.1. Participants

The participants were 417 undergraduate and students from a large public university in the southeastern United States. The sample was mostly female (*n* = 353, 85%). The sample ranged in age from 18 to 37 years, *M* = 20.8 years, *SD* = 1.9 years. The racial composition of the participant sample was: 72.9% White, 7.7% Black, 4.5%, Asian, 3.8% Latinx, 1.7% Indian subcontinent, and 9.4% multiracial or other race. Regarding political attitudes, 58.9% of the respondents indicated that they were liberal or liberal leaning and 37.1% were conservative or conservative leaning. There were 16 (3.8%) who did not indicate their political orientation. The participants completed the research as part of a research pool in the university’s College of Education in exchange for course credit. The participation in the study was completely voluntary and people had a range of alternative studies in the research pool from which to choose.

#### 4.1.2. Measures

In addition to the CCWS the participants completed four measures that included the PSWQ, FOWS, SFQ, and DASS. Researchers have used the Penn State Worry Questionnaire extensively in the past to explore its relationships with other emotions in clinical and experimental research [43]. The PSWQ consists of 16 self-report items that were designed to measure the trait of worry. People make their responses to the items on a five-point rating scale (1 to 5) with respect to how characteristic the item is of the respondent. The PSWQ is scored by summing the items; the scores range from 16 to 80. Higher scores are suggestive of clinically significant levels of worry [43,44]. The PSWQ demonstrated good test–retest reliability (*r* = 0.92) at an 8 to 10-week interval and exhibited good internal consistency (α = 0.93). For the present participants, the author observed that the PSWQ also possessed a high degree of internal consistency (α = 0.94, CI: 0.93 to 0.95).

The Storm Fear Questionnaire is a 15-item self-report measure of “the cognitive, affective, and behavioral aspects thought to be associated with storm phobia in adults.” ([21], p. 114) The authors conceptualized storms to encompass a variety of severe or extreme weather events such as thunder, lightning, hurricanes, and tornadoes, although the items only refer to storms in the SFQ. People respond to the SFQ items using a five-point rating scale (0 to 4) to indicate the extent to which each item is true for them. The total score on the SFQ are obtained by summing the item ratings; Scores range from 0 to 60 with higher scores indicating progressively greater degrees of storm-related fear and incapacity resulting from such fear. The SFQ items demonstrated very good internal consistency (α = 0.95). The two-week test–retest reliability was acceptable (*r* = 0.78). With the sample of participants in this project, the author observed that the SFQ possessed good internal consistency (α = 0.94, CI: 0.93 to 0.95).

The Fear of Weather Scale contains 65 self-report items that assess peoples’ level of fear to various types of weather (e.g., thunderstorms) and the sensory components of that weather (roaring sounds of wind). People indicated their fear of each weather component by using a 0 (No fear) to 6 (Terror) fully-anchored rating scale [22]. The item ratings are summed to yield a total score, which can range from 0 to 390; higher scores indicate greater fear of weather. The FOWS also possesses 22 items to assess the effects and consequences of severe weather on people with respect to property damages, loss of control over belongings, and loss of services (e.g., water, electricity). The FOWS exhibited good internal consistency in prior research [22]. Within the current sample of this study, the internal consistency of the 65 items assessing fear of weather was quite higher (α = 0.98, CI: 0.97 to 0.98).

The Depression, Anxiety, and Stress Scale is a 42-item measure designed to assess the emotional states rather than traits [90,91]. Each subscale consists of 14 items that measure recent characteristics of depressive emotions (gloomy outlook, pessimism, self-disparaging, disinterest, lack of motivation, etc.), anxiety (apprehensiveness, trembly, autonomic symptoms of arousal, worry about performance and loss of control), and stress (tension, easily upset, irritable, difficulty relaxing, easily startled). The DASS instruction set asks people to respond to the items using the timeframe of “over the past week” and a four-point rating scale that ranges from 0 (“Did not apply to me at all”) to 3 (“Applied to me very much or most of the time”). The DASS has seen extensive use in experimental and clinical research as evidenced by over 400 studies that have used the measure within the PsycINFO database. Most recently, the shorter version of the DASS (i.e., DASS-21) was favorably evaluated for its psychometric properties and its robustness against patient self-report bias [92]. The DASS has demonstrated stability through confirmatory factor analyses and also good levels of internal consistency (α’s ranging from 0.84 to 0.91) [90]. For the present sample, the three DASS subscale exhibited strong internal consistencies: anxiety (α = 0.94, CI: 0.93 to 0.95), depression (α = 0.96, CI: 0.95 to 0.97), and stress (α = 0.95, CI: 0.94 to 0.95).

#### 4.1.3. Procedure

The participants enrolled in the study online and then were directed first to the informed consent document. The measures of the project were administered through the Qualtrics survey platform. If the participants gave their consent for the study, then the Qualtrics platform presented the project measures in random order. The demographic items always appeared last in the survey, followed by a debriefing statement. The project measures and procedure were reviewed and approved by the Institutional Review Board at the author’s university (Protocol ID#: STUDY00006902).

#### 4.1.4. Data Analysis

The author used the R statistics package to calculate the descriptive statistics and the Pearson correlations to investigate the hypothesized relationships in this study. Then, the Lavaan package in R was used to conduct the path analysis to assess the contributions of political orientation, fear of weather, fear of storms, and stress upon CCWS scores [74].

### 4.2. Results

The correlations of the CCWS with the with the other measures in Study 3 appear in Table 7. As hypothesized, although the scores on the CCWS and the PSWQ were correlated to a statistically significant extent, the correlation was comparatively small in magnitude. This result suggested that the type of general and broad worry that characterizes high scorers on the PSWQ does not contribute in a noteworthy way to the specific worry people expressed about climate change, and thus supports the instrument’s divergent validity Similar to the results from Study 1, political orientation was related in a statistically significant and noteworthy way with CCWS scores. Again, an increasingly democratic, liberal political orientation was associated with increasing levels of climate change worry. Although women (*M* = 24.8) did produce CCWS scores that were numerically higher than men (*M* = 23.0) as observed in Study 1, the differences within the present study were not statistically significant (*p* = 0.17). This result may have been due to the fact that there were fewer men (*n* = 64) in this study compared to Study 1.

Regarding weather, both the SFQ and the FOWS exhibited comparable and statistically significant associations with scores on the CCWS. For each weather measure, increasing fears of storms or of weather elements that could be severe or extreme were associated with increases in climate change worry.

The DASS subscales each demonstrated statistically significant associations with scores on the CCWS. Increases in scores across each of the three subscales (i.e., stress, anxiety, and depression) were associated with increases in climate change worry. Contrary to what was expected, the three DASS measures exhibited comparable correlations with the CCWS. The Stress subscale, however, exhibited the most robust correlation with the CCWS as evidenced by a partial correlation analysis. After controlling for the association of the DASS anxiety subscale, the correlation of Stress with CCWS remained statistically significant, *r_Stress,CCWS|Anxiety_* = 0.12, *p* = 0.014. A similar result was observed when controlling for the DASS depression subscale: *r_Stress,CCWS|Depression_* = 0.12, *p* = 0.016. When controlling for the associations of the Stress subscale, the CCWS correlations with the Anxiety and depression subscales were not statistically significant. These results suggested that the emotional state of stress was more strongly related to climate change worry.

**Figure 4:**
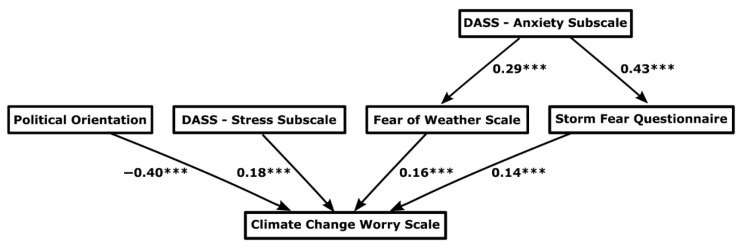
Path analysis model predicting scores on the Climate Change Worry Scale. Note: Values displayed are standardized regression coefficients. *** *p* ≤ 0.001, and R^2^_adj_ = 0.32.

The contributions of the variables to the prediction of climate change worry are depicted in the path diagram in Figure 4. This model exhibited a good fit to the data, as indicated by a comparative fit index (CFI) of 0.98 and a Tucker–Lewis index of 0.96. Similarly, both the RMSEA (0.056) and the standardized room mean square residual (0.021) were small and suggestive of a good model fit. Feelings of state anxiety (DASS anxiety) were predictive of scores on the FOWS and the SFQ; anxiety was somewhat more related with fear of storms. In turn, the FOWS and the SFQ were comparably predictive of CCWS scores. A stressful emotional state (DASS stress) also was predictive of increasing CCWS scores as was a progressively more liberal or democratic political orientation. Together, the predictive measures explained 32% of the variance in CCWS scores. Scores on the PSWQ and participant gender were not significantly predictive of scores on the CCWS.

### 4.3. Discussion

The results of this study document the convergent and divergent validity of the CCWS. The small but significant correlation between the CCWS and the PSWQ likely relates to the fact that both instruments are measures of worry (i.e., both measures use the word, worry, in their respective items). Further, because the CCWS was specifically designed to measure climate change-related worry, it is understandable that the correlation with the PSWQ is small given the more general and broad scope of the latter.

Fear of weather (FOWS) and fear of storms (SFQ) both exhibited a moderate degree of association with climate change worry, a result which supported the convergent validity of the CCWS. These results are consistent with the fact that a disrupted global climate system may lead to more severe weather outbreaks or more frequent extreme events [1]. People with existing fears of weather or storms may thus worry increasingly about climate change. This may especially be the case if people have memories or recollection of severe weather and have noted trends towards greater severity in the weather over time.

Research in the field of worry suggested that stress would relate more strongly to worry than other affects like anxiety or depression [45,46,49]. Although the three DASS measures exhibited correlations of comparable magnitudes with the CCWS, only the DASS stress subscale exhibited the most robust correlation with climate change worry. DASS anxiety was more predictive of fears of severe weather elements and fear of storms than climate change worry. This result makes sense in that the general apprehensiveness about future events (here, weather and storms) may amplify peoples’ fears of extreme weather events.

Political orientation was the most predictive of climate change worry. This result is consistent with the higher degree of correlation among these variables reported in Study 1 above and also in line with prior research results that have documented a liberal–conservative difference in beliefs about the realities of climate change, the causes of climate change, and policies to address it [18,26,66]. This result also raises the interesting question of what else may contribute to climate change worry that could help to change a person’s beliefs and attitudes so that they are more prepared to behave more sustainability and thus to mitigate global climate disruption and adapt to its varied impacts.

## 5. General Discussion

Global climate change may affect peoples’ behaviors (e.g., move from a flood plain or coastal region), their thoughts or attitudes (e.g., accept or deny the reality of climate change) and can lead to the experience of emotions such as anxiety, fear, or worry [13,23,24]. Within this article, the author reported on the initial development of a measure of worry about climate change.

The author chose to assess climate change worry rather than other affects such as fear or anxiety for three reasons. First, worry is a component of anxiety along with somatic arousal [55]. Thus, worry is a more fundamental psychological feature and one that is rooted in the symbols of thought and language [45,54,56]. Although people may typically know that they worry, how much they worry, or what they worry about so that they can self-report these experiences accurately, such may not be the case in self reports of the somatic arousal that underlies anxiety. Self-reports of arousal may not correspond to arousal measured psychophysiologically [93]. In addition, people may be differentially sensitive to interoceptive cues of their arousal [94]. Second, and relatedly, with its foundation in language and thought, worry about climate change is salient at this time when messages about climate change appear frequently within the media. From 2017 to 2019, climate change and the occurrences of severe and extreme weather have resulted in nearly 34,000 print and online articles in over 200 newspapers worldwide and approximately 3000 television and stories according to the Ebsco Newspaper Source Plus database. If people have not experienced climate change directly, then there is an increasing likelihood that they will have learned about it through the media [20]. Third, fear pertains to the anxiety and apprehension that comes with an imminent threat [51]. Because climate change may be unrealized for some people, the assessment of climate change fear may be less informative at this point, but certainly should be considered for the future [95,96]. In this regard, fear of weather or storms that people experienced in the past or anticipate for the future may relate to climate change worry [1,21,22].

Beyond these conceptual considerations, the three studies in this article were informative in the creation of a new measure of climate change worry. The ten CCWS items exhibited unidimensionality and invariance at the residual level for men and women in Study 1. In addition, a Rasch model was developed that further supported the unidimensionality of the items and that illustrated the relationship of the participants and the CCWS items with respect to their level of climate change worry (Figure 2). The results from Study 2 suggested that over a two-week test–retest interval that scores on the CCWS were both highly correlated and that the scores on the measure did not change significantly with time. The results from study 3 supported the validity of the CCWS by examining its relationship with other established measures. Although the CCWS was correlated with the PSWQ, the magnitude of the correlation was small. Climate change worry was much more related to the state-levels of stress (DASS stress), which was expected on the basis of theory [13,18,19]. Similarly, the CCWS correlations with fear of weather (FOWS and SFQ) were expected and observed given the general relationships of worry with fear [42]. Following from the results of previous research, it was not surprising that climate change worry was associated with political orientation (studies 1 and 3) [18,26,66].

This research is limited in two significant ways, the first of which pertains to the author’s choice to assess personal worry about climate change rather than a more encompassing one that concerned inter-group conflicts and/or resource scarcity [7,8,9,10,11]. Thus, the CCWS and the research reported here represents a first step to assess and quantify climate change worry. Subsequent development efforts with the scale will focus on the societal and regional contributions to climate change worry. A second limitation concerns the reliance upon university student samples from the southeastern United States in the three studies. People who are older, who live in different geographical or climatic regions, or who have a different socioeconomic status compared to students may provide responses to the CCWS that differ from the patterns observed and reported in this project. With these caveats in mind, the student samples provided an important starting point for the measurement of climate change worry given that the university is situated in an area that regularly experiences extreme of heat, flooding, severe thunderstorms and tornadoes, and that is susceptible to hurricanes or tropical storms that make landfall in the southeastern United States. In addition, the responses of student participants are valuable insofar as they reflect the experiences of people who will study, document, enact policies, and live with the results of climate change in the future.

Future research with the CCWS could occur along several lines, the first of which involves examining the functionality of the instrument with a diverse sample that is more representative of the racial and socioeconomic composition of residents in the United States. Second, the researcher will explore the development of additional items that relate to more distal and global impacts of climate change. Third, additional research should be conducted to explore the interrelationships of fear, anxiety, depression, and worry within the realm of climate change. Although the distinctions between these constructs may be somewhat clearer in the clinical psychology literature [11,12,13,14,15,16,17,18,19,20,21,22], the nature of the constructs and their interrelations within climate psychology warrants further theoretical and empirical research [25]. Specifically, can climate change with its multiple effects and impacts by themselves lead to anxiety and depressive disorders? Alternatively, do the effects of climate change contribute to the worsening or longer duration of incipient and existing disorders? Perhaps both scenarios are possible [13]. Subsequent research will hopefully bring clarity to these questions. The CCWS may be one among several tools available to researchers to use for this purpose.

## 6. Conclusions

The research in this article provided support for the psychometric properties of a new measure for worry about climate change. As a constituent of both feelings of depression and anxiety, worry is a basic psychological component. The Climate Change Worry Scale items exhibited a high degree of internal consistency and were strongly related to a single factor representing climate change worry; the structure of this factor was invariant for men and women in the sample. Peoples’ responses to the CCWS items over a two-week test–retest interval were acceptably stable. Finally, the experience of worry about the climate was related to feelings of stress, fear of storms and other severe weather features, and to a democratic/liberal political orientation. The CCWS was less related to a trait measure of worry (PSWQ) and to experiences of anxiety or depression.

## Figures and Tables

**Table 1:** Items of the Climate Change Worry Scale.

Item
1. I worry about climate change more than other people [52].
2. Thoughts about climate change cause me to have worries about what the future may hold [43,44,45,54].
3. I tend to seek out information about climate change in the media (e.g., TV, newspapers, internet) [53].
4. I tend to worry when I hear about climate change, even when the effects of climate change may be some time away [43,44,45,54].
5. I worry that outbreaks of severe weather may be the result of a changing climate [54].
6. I worry about climate change so much that I feel paralyzed in being able to do anything about it [43].
7. I worry that I might not be able to cope with climate change [43,44,45].
8. I notice that I have been worrying about climate change [43].
9. Once I begin to worry about climate change, I find it difficult to stop [43,44,45,52].
10. I worry about how climate change may affect the people I care about [44].

Note: The references following each item pertain to aspects of worry from the research literature that informed the item’s development. The instructions for the Climate Change Worry Scale are: Read each statement and indicate how frequently each statement applies to you. Respond in terms of how you generally feel. There are no right or wrong answers. The rating scale for indicating the frequency of the experience described by each item is as follows: 1 = Never; 2 = Rarely; 3 = Sometimes; 4 = Often; 5 = Always.

**Table 2:** Polychoric correlations of the Climate Change Worry Scale items (*n* = 600).

CCWS Item	1	2	3	4	5	6	7	8	9	10
1. I worry about climate change more than other people.	--									
2. Thoughts about climate change cause me to have worries about what the future may hold.	0.86	--								
3. I tend to seek out information about climate change in the media (e.g., TV, newspapers, internet).	0.75	0.70	--							
4. I tend to worry when I hear about climate change, even when the effects of climate change may be some time away.	0.84	0.87	0.78	--						
5. I worry that outbreaks of severe weather may be the result of a changing climate.	0.73	0.75	0.65	0.80	--					
6. I worry about climate change so much that I feel paralyzed in being able to do anything about it.	0.71	0.72	0.62	0.72	0.67	--				
7. I worry that I might not be able to cope with climate change.	0.65	0.68	0.52	0.64	0.62	0.79	--			
8. I notice that I have been worrying about climate change.	0.78	0.81	0.72	0.81	0.72	0.80	0.80	--		
9. Once I begin to worry about climate change, I find it difficult to stop.	0.67	0.66	0.58	0.66	0.59	0.77	0.77	0.81	--	
10. I worry about how climate change may affect the people I care about.	0.73	0.77	0.66	0.76	0.71	0.69	0.70	0.81	0.72	--
Mean	2.98	3.22	2.96	3.19	3.24	1.93	2.12	2.63	2.01	2.84
Variance	1.47	1.56	1.46	1.67	1.76	1.31	1.35	1.52	1.32	1.81
Skewness	−0.13	−0.31	−0.05	−0.23	−0.30	1.16	0.80	0.14	0.97	0.06
Kurtosis	−0.95	−0.89	−0.89	−1.02	−1.05	0.45	−0.29	−1.06	0.05	−1.14
Item to total (Pearson)	0.86	0.87	0.77	0.88	0.81	0.78	0.77	0.90	0.76	0.84

Note: All polychoric correlation coefficients among the items were statistically significant, *p* < 0.0001. Pearson correlations were calculated for the item-to-total correlations. All of the item-to-total correlations were statistically significant, *p* < 0.0001. The correlations and descriptive statistics are based upon the responses of 300 men and 300 women. There were no missing data.

**Table 3:** Item factor loadings and communalities.

CCWS Item	Factor Loading	Communality
1	0.853	0.728
2	0.862	0.744
3	0.741	0.549
4	0.873	0.761
5	0.777	0.603
6	0.748	0.560
7	0.738	0.545
8	0.896	0.804
9	0.729	0.532
10	0.820	0.672

**Table 4:** Indices of measurement invariance of the CCWS for women and men.

Constraint (Invariance)	df	χ^2^	Δχ^2^	Δdf	*p* χ^2^	CFI	RMSEA	ΔCFI	ΔRMSEA
None (Configural)	70	228.66	--	--	--	0.992	0.099	--	--
Loadings (Metric)	79	282.69	13.946	9	0.124	0.991	0.097	0.001	0.002
Intercepts (Scalar)	88	292.99	18.967	9	0.025	0.991	0.093	0.000	0.004
Residuals (Residual)	98	319.92	14.823	10	0.139	0.991	0.090	0.000	0.003
Means	99	485.93	16.506	1	0.000	0.985	0.115	0.006	0.025

**Table 5:** Response category statistics and Rasch–Andrich thresholds.

Item Score	Response Category Name	Item Score Frequency (%)	Average Meas.	Expected Meas.	Outfit	Rasch–Andrich Threshold
1	Never	21%	−3.27	−3.21	1.0	
2	Rarely	22%	−1.41	−1.47	0.9	−2.34
3	Sometimes	25%	−0.13	−0.12	1.0	−0.90
4	Often	22%	1.20	1.15	1.1	0.65
5	Always	11%	2.65	2.74	1.2	2.58

**Table 6:** Item characteristics and Rasch model fit statistics

CCWS Item	Construct Level	Model SE	Discrim.	Infit Mn. Sq.	Outfit Mn. Sq.
1. I worry about climate change more than other people.	−0.60	0.06	1.20	0.77	0.84
2. Thoughts about climate change cause me to have worries about what the future may hold.	−1.10	0.06	1.26	0.76	0.75
3. I tend to seek out information about climate change in the media (e.g., TV, newspapers, internet).	−0.55	0.06	0.58	1.29	1.52
4. I tend to worry when I hear about climate change, even when the effects of climate change may be some time away.	−1.05	0.06	1.28	0.74	0.77
5. I worry that outbreaks of severe weather may be the result of a changing climate.	−1.16	0.06	0.76	1.22	1.22
6. I worry about climate change so much that I feel paralyzed in being able to do anything about it.	1.76	0.07	0.99	1.10	0.90
7. I worry that I might not be able to cope with climate change.	1.29	0.06	0.80	1.23	1.14
8. I notice that I have been worrying about climate change.	0.15	0.06	1.42	0.62	0.66
9. Once I begin to worry about climate change, I find it difficult to stop.	1.56	0.06	0.83	1.21	1.30
10. I worry about how climate change may affect the people I care about.	−0.31	0.06	0.92	1.11	1.07

**Table 7:** Correlation of the Climate Change Worry Scale with political orientation, fear of weather/storms and other negative emotion.

Measure	1	2	3	4	5	6	7	8
1. Climate Change Worry Scale	--							
2. Political Orientation	−0.43 **	--						
3. Fear of Weather Scale—Total	0.30 **	−0.03	--					
4. Storm Fear Questionnaire—Total	0.30 **	−0.07	0.52 **	--				
5. DASS Stress Subscale	0.31 **	−0.10 *	0.33 **	0.38 **	--			
6. DASS Anxiety Subscale	0.29 **	−0.08	0.30 **	0.44 **	0.84 **	--		
7. DASS Depression Subscale	0.30 **	−0.13 *	0.27 **	0.37 **	0.84 **	0.83 **	--	
8. Penn State Worry Questionnaire	0.17 *	−0.02	0.24 **	0.17 *	0.47 **	0.33 **	0.30 **	--
Mean	24.43	3.11	173.0	23.45	26.22	22.59	22.73	53.48
Standard Deviation	9.47	1.57	54.80	11.18	9.64	8.86	9.66	13.77

Note: ** *p* < 0.0001; * *p* < 0.05.

## Data Availability

Contact the author with questions or requests regarding the data in this project.
